# SARS-CoV-2 Surveillance in Free-Ranging Wildlife in New England and Virginia, United States, 2022–2025

**DOI:** 10.3390/v18080832

**Published:** 2026-07-29

**Authors:** Idrissa Nonmon Sanogo, Wendy B. Puryear, Alexa F. Simulynas, Elena Cox, Maureen Murray, Zain Khalil, Harm van Bakel, Martin J. R. Feehan, Zak Mertz, Priya Patel, Jennifer Riley, Blaine Hymel, Jonathan A. Runstadler

**Affiliations:** 1 Department of Infectious Disease and Global Health, Cummings School of Veterinary Medicine, Tufts University, North Grafton, MA 01536, USA; 2 Department of Genetics and Genomic Sciences, Icahn School of Medicine at Mount Sinai, New York, NY 10029, USA; 3 Department of Microbiology, Icahn School of Medicine at Mount Sinai, New York, NY 10029, USA; 4 Department of Artificial Intelligence and Human Health, Icahn School of Medicine at Mount Sinai, New York, NY 10029, USA; 5 Massachusetts Division of Fisheries and Wildlife, Westborough, MA 01581, USA; 6 New England Wildlife Center, Weymouth, MA 02190, USA; 7 Blue Ridge Wildlife Center, Boyce, VA 22620, USA; 8 Wildlife Clinic of Rhode Island, Saunderstown, RI 02874, USA

**Keywords:** SARS-CoV-2, wildlife, surveillance, spillover, New England, Virginia, United States

## Abstract

Since its emergence in 2019, severe acute respiratory syndrome coronavirus 2 (SARS-CoV-2) has infected a wide range of animal species, including wildlife. Although SARS-CoV-2 infection has been widely reported in wildlife, particularly in white-tailed deer (WTD; *Odocoileus virginianus*) across the United States, data on viral circulation in New England wildlife remain limited. Here, we investigated active SARS-CoV-2 infection and serological evidence of previous exposure in free-ranging wildlife from New England and Virginia. We examined samples from 1646 animals representing 29 wildlife species, collected through wildlife rehabilitation centers, clinics, and hunter harvests in New England and Virginia between 2022 and 2025. SARS-CoV-2 RNA was detected in three WTD from Massachusetts and Vermont. Phylogeographic analysis showed that the Vermont WTD SARS-CoV-2 sequences were closely related to contemporaneous human SARS-CoV-2 sequences from the same region, consistent with a possible human-to-deer spillover event. Serological screening by ELISA detected SARS-CoV-2 reactive samples in nine individuals from three species, including Eastern cottontail (*Sylvilagus floridanus*), Eastern coyote (*Canis latrans*), and raccoon (*Procyon lotor*), providing putative evidence of prior SARS-CoV-2 exposure. However, neutralizing antibodies against the SARS-CoV-2 Omicron variant were detected in only a single Eastern cottontail. Overall, these findings indicate sporadic SARS-CoV-2 detection and limited serological evidence of prior exposure among wildlife sampled in New England and Virginia, and highlight the importance of continued surveillance to detect spillover events, monitor viral evolution, and assess the potential risks associated with wildlife reservoirs.

## 1. Introduction

Severe Acute Respiratory Syndrome Coronavirus 2 (SARS-CoV-2), the causative agent of the COVID-19 pandemic, has spread through human-to-human transmission since it emerged in late 2019. However, in addition to humans, many domestic and wild animal species have been reported to be either naturally infected with SARS-CoV-2 [1,2,3,4,5,6] or susceptible to the virus following experimental infection [1,7,8,9,10]. As of September 2025, SARS-CoV-2 has been detected in 68 animal species across 48 countries, highlighting the broad spectrum of the virus and its remarkable ability to infect new animal species [11].

In the United States, SARS-CoV-2 infection in free-ranging wildlife has been sporadic and confirmed in only nine species, including white-tailed deer (WTD), Eastern cottontail, Virginia opossum, deer mice, Eastern red bat, raccoon, groundhog, wild American mink, and mule deer [2,12]. Of note, aside from a single reported infection in an Eastern red bat [2], SARS-CoV-2 has not been detected in wild North American bats, and both little brown bats and big brown bats appeared to be resistant to infection [13,14,15]. In addition to SARS-CoV-2 infection, serological evidence of SARS-CoV-2 exposure has been reported in coyote, muskrat, Eastern gray squirrel, and white-footed mouse [2,16]. However, to date, WTD remain the only free-ranging wildlife species with widespread exposure to SARS-CoV-2 and are considered capable of sustaining within-species transmission with the potential for spillback to humans [6,12,17,18,19,20,21,22,23,24].

Since its emergence, numerous SARS-CoV-2 variants have been detected in humans and an increasing number of animals, raising concerns about whether free-ranging wildlife species, especially those living in urban and peri-urban areas, may also be infected through spillover from humans [2,18,19,22]. The establishment of wildlife species as reservoir hosts, which could potentially propagate new SARS-CoV-2 variants and facilitate onward transmission to humans and other animals, remains a concern [2,18]. Consequently, understanding the epidemiology and evolutionary dynamics of SARS-CoV-2 in wildlife is important for assessing potential human–animal transmission and monitoring the emergence of novel variants that may spill back into human populations [21].

Multiple studies across the United States examining the circulation of SARS-CoV-2 in free-ranging wildlife have shown that transmission dynamics and prevalence vary significantly across regions, reflecting differences in local epidemiological and ecological conditions [2,25,26,27,28,29,30]. However, data on SARS-CoV-2 circulation in New England wildlife are limited, and the impact of the virus on local wildlife health is unclear. These findings collectively emphasize the need for ongoing surveillance to identify potential wildlife hosts that could harbor and spread the virus, support viral evolution, and facilitate possible spillback to humans.

Wildlife rehabilitation centers (WRCs) and wildlife clinics provide access to a diverse range of understudied species that can be challenging to sample in the field. They also provide opportunities for frequent human–animal interactions, potentially increasing the risk of spillover, making WRCs particularly valuable for pathogen surveillance, including SARS-CoV-2 [31].

In this study, we investigated SARS-CoV-2 infection and serological evidence of past exposure in 29 wildlife species, either admitted to rehabilitation centers and wildlife clinics or obtained through hunter harvests in New England and Virginia between 2022 and 2025, to better understand the prevalence of SARS-CoV-2 in free-ranging wildlife.

## 2. Materials and Methods

### 2.1. Sample Collection

**Figure 1:**
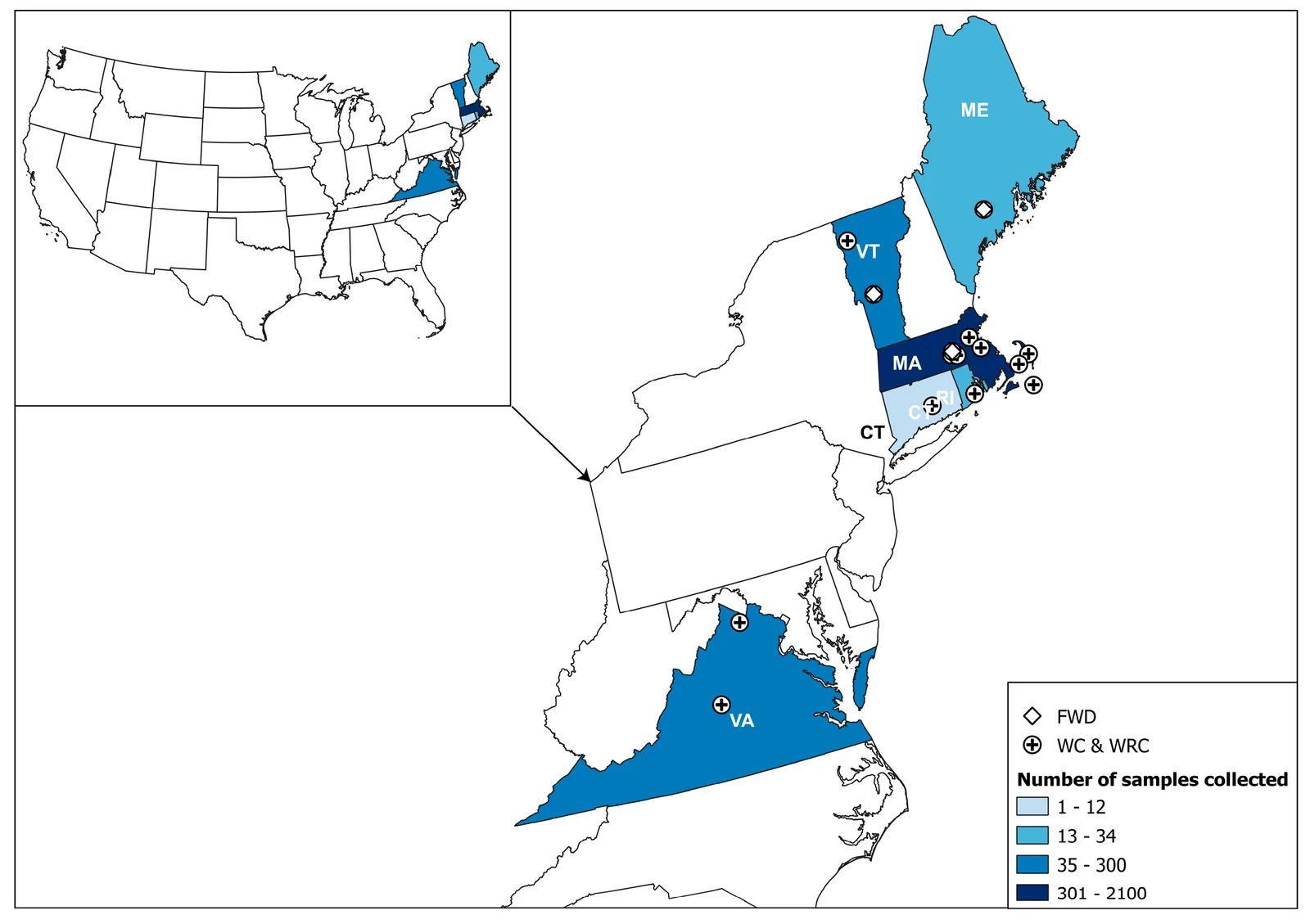
Map showing sample collection sites for SARS-CoV-2 surveillance in New England and Virginia, from 2022 to 2025. Color intensity indicates the relative number of samples collected per state (CT: Connecticut, ME: Maine, MA: Massachusetts, RI: Rhode Island, VT: Vermont, VA: Virginia). Symbols denote the locations of State Fish and Wildlife Agencies (FWD), wildlife clinics (WC), and wildlife rehabilitation centers (WRC) that contributed samples.

From 2022 to 2025, we leveraged our ongoing surveillance efforts through a network of wildlife clinics, rehabilitation facilities, and State Fish and Wildlife Agencies across New England and Virginia to collect 2053 nasal, oropharyngeal, and rectal swab samples, as well as 502 serum samples from 1646 animals representing 29 peri-urban wildlife species (Table 1). A subset of the samples (314 nasal and oropharyngeal swabs and 40 serum samples) was collected from hunter-harvested white-tailed deer at check stations in Vermont and Massachusetts in 2024. 80.2% (*n* = 1647) of the swabs and 88.8% (*n* = 446) of the serum samples were collected in Massachusetts, and the remaining samples were collected in Connecticut, Maine, Rhode Island, Vermont, and Virginia (Figure 1). Each center received sampling supplies along with standard operating procedures for collecting, storing, and shipping samples to the laboratory.

Swabs were placed in individually labeled sterile vials containing 700 µL of viral transport media [32] and stored at −20 °C in the rehabilitation facilities until shipped to the laboratory. In the laboratory, all swab specimens were stored at −80 °C, and serum samples were maintained at −20 °C until testing. Metadata for each animal tested for SARS-CoV-2, including species name, animal ID, sample type, collection date, location, and the facility that collected the samples, are provided in Supplementary Table S1.

### 2.2. RNA Extraction and SARS-CoV-2 RT-qPCR

RNA was extracted from swabs using the Mag-Bind Viral DNA/RNA Extraction Kit (cat. M6246-02, Omega Bio-Tek, Norcross, GA, USA) on a KingFisher Flex platform (Thermo Fisher Scientific, Waltham, MA, USA), following the manufacturer’s protocol. All samples were tested for SARS-CoV-2 viral RNA by semi-quantitative real-time reverse transcription PCR (rRT-PCR) on a StepOnePlus platform (ABI). The assay was performed as previously described [33] using qScript XLT 1-Step RT-PCR ToughMix (Cat. No. 95132-500, Quantabio, Beverly, MA, USA) with two primer sets: one targeting the SARS-CoV-2 ORF1b gene and another for the internal control (β-actin). The positive Cq cutoff value was set at 40 cycles. Each plate included viral transport medium (VTM) as a negative control and a positive control (Genomic RNA from SARS-CoV-2, isolate USA-WA1/2020, BEI Resources, Manassas, VA, USA). Samples that failed to amplify β-actin in the internal control assay were considered to contain insufficient host RNA and were excluded from SARS-CoV-2 prevalence estimation. Animals were included in the prevalence estimates if at least one sample from the individual successfully amplified β-actin. Following initial SARS-CoV-2 RNA detection, positive samples were submitted to the National Veterinary Services Laboratories (NVSL) for confirmation.

### 2.3. SARS-CoV-2 Genome Sequencing and Phylogeographic Analyses

RNA extracted from positive specimens was subjected to cDNA synthesis using ProtoScript II (cat. E6560, New England Biolabs, Ipswich, MA, USA), followed by whole-genome amplification with two custom and interleaved primer panel sets targeting 1.5 and 2 kb overlapping regions across the SARS-CoV-2 genome, as previously described [34]. PCR amplification was done with Q5 Hot Start High-Fidelity DNA polymerase (cat. M0493, New England Biolabs, Ipswich, MA, USA) in a 25 μL reaction volume. Amplicons were then purified using a 1.8X ratio of Ampure XP beads (cat. A63882, Beckman Coulter, Indianapolis, IN, USA) and verified by gel electrophoresis. Next, we performed library preparation using the Nextera XT kit (cat. FC-131-1096, Illumina, San Diego, CA, USA) followed by paired-end sequencing (2 × 150 nt) on the Illumina MiSeq platform. SARS-CoV-2 genomes were assembled and subjected to quality control using the vRAPID pipeline [35]. Genotypic analysis and clade/lineage assignment of complete genomes were performed using Nextclade CLI (v3.12.0) and pangolin (v4.3) [36,37].

For the phylogeographic analysis, we compiled a dataset of SARS-CoV-2 sequences from 2023 to 2024, including all publicly available deer sequences from GISAID and BV-BRC, human sequences from Vermont and neighboring states (Massachusetts, New Hampshire, and New York), and the 50 most similar sequences identified through a BLAST (version 2.17.0) search in GenBank. Whole-genome sequences were aligned using MAFFT v7.526 [38], redundant sequences were removed with CD-HIT v4.8.1 [39], and alignments were manually inspected in BioEdit 7.2.5 [40]. We inferred a maximum-likelihood phylogeny with IQ-TREE v2.4 [41], using ModelFinder [42] to choose the best-fitting nucleotide substitution model and to identify clades corresponding to independent introductions of SARS-CoV-2 lineages into Vermont white-tailed deer. The resulting phylogenetic tree was used to downsample the dataset for subsequent analyses by excluding outgroup sequences.

A total of 105 sequences were used for subsequent phylogenetic and evolutionary analyses. To investigate the evolutionary history and estimate the time to the most recent common ancestor of the SARS-CoV-2 sequences generated in this study, we performed a discrete phylogeographic reconstruction using a Bayesian Markov Chain Monte Carlo (MCMC) approach in BEAST 1.10.4 [43]. We used the GTR substitution model with gamma-distributed rate variation among sites with a strict molecular clock model. MCMC chains were run until convergence was reached for all parameters (effective sample size (ESS) > 200). After discarding 10% of the sampled trees as burn-in, a maximum clade credibility (MCC) tree was summarized using TreeAnnotator v1.10.4 and visualized using the ggtree (v3.8.2) package in R.

### 2.4. SARS-CoV-2 Antibody Detection by ELISA

Serum samples were tested in duplicate for antibodies against the SARS-CoV-2 RBD using an in-house ELISA for assessing SARS-CoV-2 antibodies in ferrets, with minor modifications [33]. Briefly, Immulon 4 HB plates were coated with 2 μg/mL purified SARS-CoV-2 RBD (cat. NR-52366, BEI Resources, Manassas, VA, USA) and incubated for 24 h at 4 °C. Following incubation, plates were washed three times with phosphate-buffered saline supplemented with 0.1% Tween-20 (PBS-T) and blocked with Pierce Protein-Free Blocking Buffer (cat. 37585, Thermo Fisher Scientific, Waltham, MA, USA) at room temperature for 1 h. Serum samples were diluted 1:5 in PBS on different plates, then transferred to ELISA plates at a final dilution of 1:50 in PBS-T with 1% milk. The plates were then incubated at room temperature for 2 h. Positive controls consisted of serum from spike protein-immunized alpacas. After incubation, plates were washed three times, and 50 μL of Pierce Recombinant Peroxidase Conjugated Protein A/G (cat 32490, Thermo Fisher Scientific, Waltham, MA, USA) was added at 1:10,000 in PBS-T containing 1% milk and incubated for 1 h at room temperature. Plates were then washed and developed with SigmaFast o-phenylenediamine dihydrochloride tablets (cat. P9187, Sigma-Aldrich, St. Louis, MO, USA) for 10 min, stopped with 50 μL 3M HCl, and read at 490 nm on a BioTek Synergy 4 Multidetection Plate Reader (BioTek Instruments, Winooski, VT, USA). Pre-pandemic wildlife serum samples for most of the species tested were unavailable. Therefore, eight technical replicates of the alpaca serum were included as negative controls on each ELISA plate to establish assay background and cutoff values. The positivity cutoff was set as μ + 3σ of the negative controls (*n* = 8).

Positive samples from the initial ELISA-RBD screening were validated by a confirmatory ELISA using 3-fold serial dilutions starting at 1:100 against the full-length SARS-CoV-2 spike protein (BEI Resources, NR-52308) [44]. Spike ELISA-reactive samples were defined as samples showing a signal greater than the mean plus three standard deviations (μ + 3σ) of the negative controls in at least the first two consecutive dilutions.

### 2.5. SARS-CoV-2 Neutralization Assay

We performed a SARS-CoV-2 virus neutralization test (VNT) in our BSL-3 facility at the New England Regional Biosafety Laboratory (NERBL) to assess neutralizing antibodies in ELISA-positive sera [45]. Serum samples were heat-inactivated (56 °C, 30 min) and serially two-fold diluted starting at 1:4, in duplicate. Dilutions were incubated with 200 TCID_50_ of SARS-CoV-2 Omicron (isolate HCoV-19/USA/MDHP20874/2021; BEI Resources NR-56461) or the ancestral SARS-CoV-2 strain (USA-WA1/2020; cat. NR-52281, BEI Resources, Manassas, VA, USA) for 1 h at room temperature. The Omicron variant was used because Omicron-lineage viruses were the predominant SARS-CoV-2 strains circulating in human populations during the period when most serum samples were collected (2022–2024). The virus–serum mixtures were added to confluent Vero E6 cells (ATCC, cat. CRL-1586, Manassas, VA, USA) in 96-well plates and incubated at 37 °C with 5% CO_2_. Cytopathic effect was assessed at 5 days post-infection, and the neutralization titer was defined as the highest serum dilution that achieved complete (100%) inhibition of CPE in both replicate wells. Neutralization titers ≥16 were considered seropositive. An animal was considered seropositive only if the corresponding sample tested positive by both ELISA and VNT. Animals that tested positive in both RBD and Spike ELISA and failed in neutralization assays were considered putatively seropositive to SARS-CoV-2.

## 3. Results

### 3.1. Prevalence of SARS-CoV-2 Infection in Free-Ranging Wildlife by RT-PCR

We tested a total of 2053 swabs from 1646 individual animals representing 29 wildlife species by RT-PCR. 81.5% of the swabs (*n* = 1673) tested positive for β-actin, indicating the presence of adequate host RNA. Samples that failed the internal control (*n* = 380) were excluded from SARS-CoV-2 prevalence estimates. All 159 WTD had at least one beta-actin-positive swab, and SARS-CoV-2 RNA was detected in three (1.9%) of the 159 animals tested (one out of 56 in Massachusetts and two out of 103 in Vermont). The two positive white-tailed deer samples from Vermont were confirmed by NVSL. The third sample, collected in Massachusetts, had insufficient remaining material for submission and confirmatory testing at NVSL. The SARS-CoV-2-positive deer in Massachusetts was a young fawn with skull fractures recovered from a trail in 2022 and brought to Tufts Wildlife Clinic at Cummings School of Veterinary Medicine at Tufts University (RT-PCR Cq = 35).

The two additional positive white-tailed deer were hunter-harvested in Vermont in November 2024, and both nasal and oral swabs tested positive. SARS-CoV-2 whole-genome sequences were successfully generated from the oropharyngeal swabs of both animals (Table 2). We found no detectable SARS-CoV-2 RNA within any swab samples collected from the other wildlife species.

### 3.2. Whole-Genome Sequences and Phylogeographic Analysis of SARS-CoV-2 in WTD

Pairwise comparison of the two SARS-CoV-2 genomes obtained from WTD in Vermont revealed 99.99% nucleotide identity, indicating a high degree of similarity between the sequences. The two sequences were classified within Nextstrain clade 24C and Pango lineage KP.3.2.8, and exhibited the following spike protein substitutions relative to the BA.2.86 reference strain: S12F, A27S, L212I, F456L, and Q493E (Table 2). The KP.3.2.8 lineage was among the dominant SARS-CoV-2 variants circulating among humans in many U.S. states during the time of collection in November 2024.

**Figure 2:**
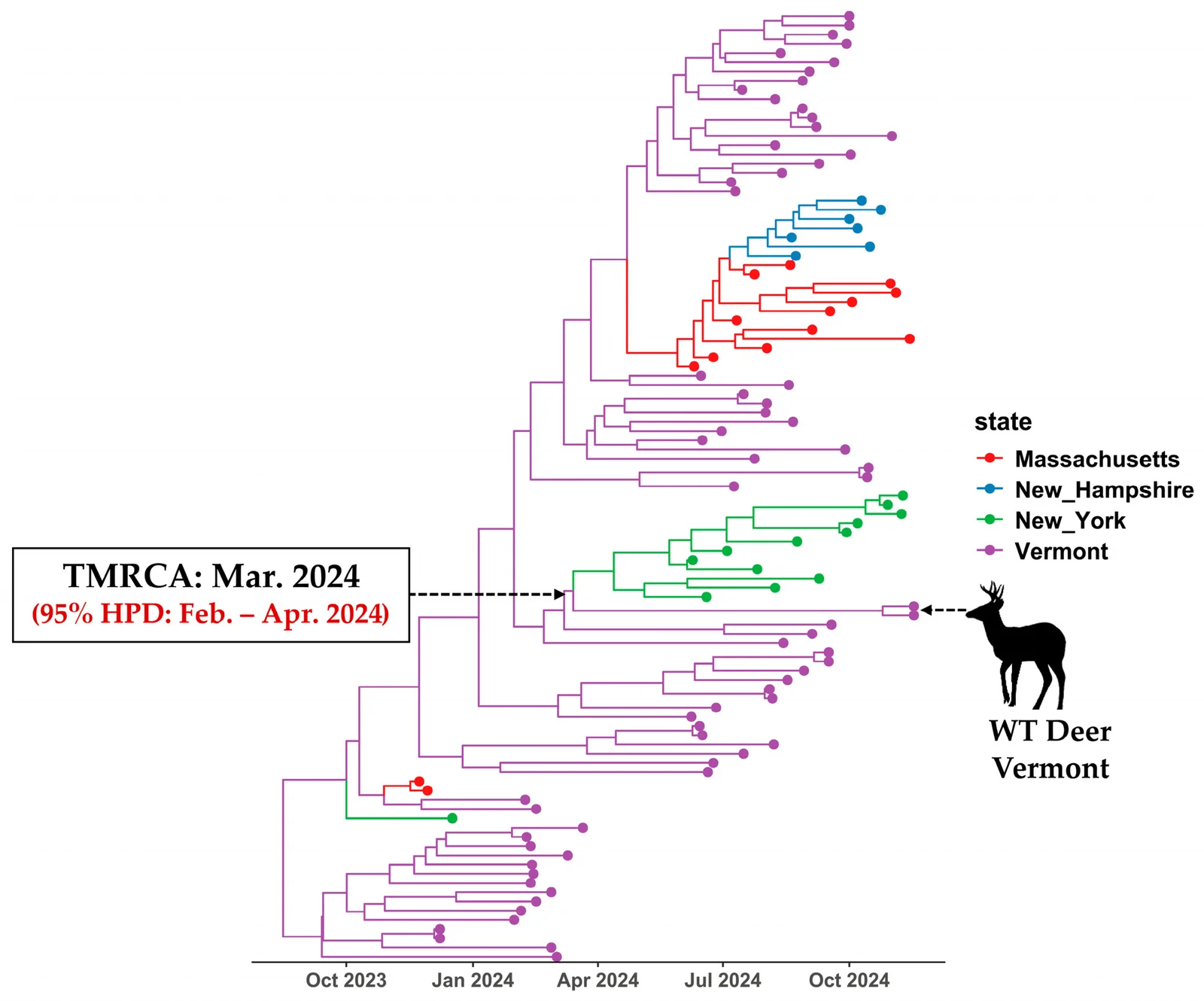
Maximum clade credibility (MCC) tree of Vermont WTD SARS-CoV-2 sequences. Branches are colored by state, with the x-axis representing the time scale. Vermont deer sequences clustered with human-derived sequences from Vermont (purple) and New York (green). The box with TMRCA indicates the time to the most recent common ancestor for the Vermont WTD sequences with its 95% highest posterior density.

We performed a time-calibrated Bayesian phylogenetic analysis to investigate the origin of SARS-CoV-2 in WTD in Vermont. The maximum clade credibility (MCC) tree showed that the two Vermont WTD sequences clustered closely with contemporaneous human sequences from Vermont and New York (Figure 2). The estimated time to the most recent common ancestor for the WTD sequences was March 2024 (95% highest posterior density [HPD]: February–April 2024). The MCC tree identified a human SARS-CoV-2 genomic sequence from Vermont as the most likely precursor of the two WTD sequences, consistent with a possible spillover event from human to WTD followed by circulation within deer (Figure 2). However, given the limited number of SARS-CoV-2 genomes available in WTD, multiple closely related spillover events cannot be excluded.

Notably, our phylogenetic analyses revealed no evidence of spillback from white-tailed deer to humans, as no human-derived genomes clustered with or were directly derived from the Vermont white-tailed deer sequences.

### 3.3. Serological Evidence of SARS-CoV-2 Infection in Wildlife

Among the 502 serum samples collected, 32 tested positive in the initial SARS-CoV-2 RBD ELISA screening. However, confirmatory testing with the SARS-CoV-2 full-spike ELISA detected antibodies in only 9 samples from three wildlife species: the Eastern cottontail (2%, *n* = 4/193), Eastern coyote (18%, *n* = 2/11), and raccoon (5.7%, *n* = 3/52). All ELISA-positive samples were collected in Massachusetts between 2023 and 2024. Among the Spike ELISA-positive samples, SARS-CoV-2–neutralizing antibodies against the omicron variant were detected in a single Eastern cottontail sampled in 2024 (Table 3). In contrast, none of the serum samples showed detectable neutralizing activity against the ancestral SARS-CoV-2 WA1/2020 strain. Further neutralization assays could not be performed on the Eastern cottontail serum sample with Omicron-neutralizing activity due to insufficient remaining volume.

## 4. Discussion

In this study, we examined the circulation of SARS-CoV-2 in 1646 free-ranging terrestrial mammals sampled in New England and Virginia between 2022 and 2025.

We detected SARS-CoV-2 RNA in 1.9% (*n* = 3/159) of white-tailed deer (WTD) sampled in New England, which is a lower prevalence compared to other states, including Ohio (35.8%), Iowa (33.2%), New York (21.1%), Pennsylvania (16.3%), and Nebraska (16.3%) [12,19,22,24,30]. However, our findings align with previous studies of SARS-CoV-2 in WTD, including surveys in Vermont that detected no infection in white-tailed deer during the 2021 and 2022 harvest seasons [25], and only 0.76% of free-ranging deer in Florida tested positive [46].

White-tailed deer are known to be susceptible to SARS-CoV-2 infection and have transmitted the virus under both natural [12,20,22,24] and experimental conditions [8,17,23]. Our phylogeographic analyses showed that the WTD SARS-CoV-2 sequences from Vermont were closely related to contemporaneous human SARS-CoV-2 sequences from the same region, consistent with a possible human-to-deer spillover event followed by local circulation in deer. However, these findings should be interpreted cautiously because only two deer-derived genomes were available for phylogenetic analysis. Consequently, multiple closely related spillover events from humans to deer cannot be ruled out.

To date, natural SARS-CoV-2 infections detected in white-tailed deer have primarily originated from spillovers from humans, with subsequent instances of deer-to-deer transmission [12,19]. The limited prevalence of SARS-CoV-2 observed in Massachusetts and Vermont deer may reflect reduced opportunities for human–deer transmission and lower SARS-CoV-2 circulation in human populations [25]. Vermont is sparsely populated and characterized by extensive rural and forested habitats, which may reduce direct or indirect contact between deer and humans. Moreover, reported human COVID-19 incidence in Vermont was markedly lower than in neighboring states during our surveillance period, which could further limit opportunities for human-to-deer spillover [47].

Compared with other U.S. states where SARS-CoV-2 has been widespread among free-ranging wildlife, including reports from Virginia between 2022 and 2023 [2], we found active infections only in white-tailed deer, not in any other sampled species. Our results are consistent with earlier reports from Vermont and various other U.S states, where active infections were either limited to white-tailed deer or not present in other wildlife species [25,26,27,28,30]. Collectively, these results suggest that SARS-CoV-2 spillover and subsequent animal-to-animal transmission likely depend on a combination of ecological conditions, human infection dynamics, and species-specific susceptibility, which may limit sustained circulation in some regions such as New England.

Bats are recognized as reservoirs for numerous zoonotic viruses, including members of the Coronaviridae family [48]. In this study, we tested 359 swabs from six bat species, with 95% of the swabs from big brown bats, to assess their potential susceptibility to SARS-CoV-2. No SARS-CoV-2 RNA was detected in any of the samples. These findings are consistent with previous experimental challenge studies, which concluded that North American bat species, including big brown and little brown bats, are unlikely to be susceptible to SARS-CoV-2 infection [14,15]. Nevertheless, it is worth noting that SARS-CoV-2 RNA was detected in one Eastern red bat, although the context of exposure remains unclear, and this species has not been evaluated in laboratory experiments [2].

Although no active SARS-CoV-2 infection was found in wildlife except in WTD, we detected serological evidence suggestive of prior exposure in eastern cottontails, coyotes, and raccoons, and evidence of past exposure with neutralizing antibodies against the SARS-CoV-2 Omicron variant in a single eastern cottontail sampled in Sutton, MA, in 2024. While the exact route of exposure remains uncertain, the presence of eastern cottontails in urban or peri-urban areas and their frequent interactions with humans or human-contaminated environments may have allowed for potential spillover [49]. Despite experimental evidence indicating limited susceptibility of eastern cottontails to earlier SARS-CoV-2 strains [50], SARS-CoV-2 RNA has been detected in eastern cottontails in Virginia [2], supporting the possibility of field exposure and suggesting that susceptibility may vary with circulating viral strains.

None of the ELISA-positive samples from coyotes and raccoons demonstrated neutralizing activity against SARS-CoV-2, which may indicate previous exposure or cross-reactive binding antibodies that do not confer functional neutralization [51]. This aligns with reports suggesting that both species have been exposed to the virus [2,16]. Notably, prior experimental challenge studies have found that coyotes and raccoons are unlikely to serve as reservoir hosts for SARS-CoV-2 [1,9]. We did not detect neutralizing antibodies against the ancestral SARS-CoV-2 WA1/2020 strain. However, most samples were collected during 2022–2024, when Omicron variants predominated in the human population, potentially limiting cross-neutralization against the ancestral virus.

While our study provides valuable insights into the prevalence of SARS-CoV-2 in free-ranging wildlife in New England and Virginia, a few limitations should be considered. First, samples were collected opportunistically, and sample sizes were limited for some species (fewer than five individuals). Additionally, most of our samples (>80%) were collected in Massachusetts, which may not reflect the full ecological diversity of wildlife in the region and may bias prevalence estimates toward species or locations that were more accessible during sampling. Second, although we collected 2053 swab samples, serum, which is more likely to give information on prior exposure, was available for only a subset of the animals tested (*n* = 502), limiting our ability to assess prior exposure for certain species. Third, serological findings, particularly those from the SARS-CoV-2 RBD ELISA, should be interpreted cautiously because species-specific negative controls were unavailable for several wildlife species included in this study. However, the use of additional confirmatory assays, including SARS-CoV-2 spike ELISA and virus neutralization testing, helped reduce the likelihood of nonspecific seroreactivity. Although the RBD ELISA has previously been applied for SARS-CoV-2 antibody detection in non-human mammals, including ferrets [33], and the secondary antibody used recognizes immunoglobulins from a broad range of mammalian species, differences in antibody binding efficiency among wildlife species may have influenced assay sensitivity. Consequently, the possibility of false-negative results cannot be completely excluded. Finally, virus neutralization testing using the Omicron variant and the ancestral SARS-CoV-2 WA1/2020 strain did not detect neutralizing antibodies in the available ELISA-positive sera, except for a single Eastern cottontail serum sample that neutralized the Omicron variant. These findings underscore the importance of interpreting wildlife serological data with caution and suggest that evidence of exposure based on binding antibody assays should not be interpreted as definitive evidence of neutralizing antibody responses.

Despite these limitations, our findings provide important baseline data on SARS-CoV-2 exposure and infection in free-ranging wildlife in New England and Virginia, highlighting the need for continuous surveillance and experimental studies to clarify species susceptibility and spillover risk.

## Figures and Tables

**Table 1:** Number and type of samples collected from wildlife.

Species	Scientific Name	Unique Animals	Swabs	Serum Samples
**Bats**				
Big brown bat	*Eptesicus fuscus*	219	342	4
Eastern red bat	*Lasiurus borealis*	3	4	-
Eastern small-footed bat	*Myotis leibii*	3	5	-
Evening Bat	*Nycticeius humeralis*	3	3	-
Little brown bat	*Myotis lucifugus*	2	2	-
Silver-haired bat	*Lasionycteris noctivagans*	2	3	-
**Carnivores**				
Black Bear	*Ursus americanus*	6	6	1
Bobcat	*Lynx rufus*	22	26	11
Eastern coyote	*Canis latrans*	20	23	11
Fisher	*Pekania pennanti*	14	16	1
Gray fox	*Urocyon cinereoargenteus*	19	20	4
Long-tailed Weasel	*Neogale frenata*	4	4	3
Raccoon	*Procyon lotor*	165	168	52
Red fox	*Vulpes vulpes*	57	72	13
River otter	*Lontra canadensis*	5	6	-
Striped skunk	*Mephitis mephitis*	64	65	22
**Rodents & Lagomorphs**				
Eastern chipmunk	*Tamias striatus*	18	25	4
Eastern cottontail	*Sylvilagus floridanus*	413	437	193
Eastern gray squirrel	*Sciurus carolinensis*	166	194	51
Groundhog	*Marmota monax*	42	44	10
Muskrat	*Ondatra zibethicus*	13	13	2
North American beaver	*Castor canadensis*	10	11	5
North American porcupine	*Erethizon dorsatum*	46	46	17
Norway rat	*Rattus norvegicus*	17	17	8
Red squirrel	*Tamiasciurus hudsonicus*	11	11	4
Southern Flying Squirrel	*Glaucomys volans*	6	12	-
White-footed mouse	*Peromyscus leucopus*	5	5	-
**Other Mammals**				
Virginia opossum	*Didelphis virginiana*	132	139	34
White-tailed deer (WTD) *	*Odocoileus virginianus*	159	334	51
Total		1646	2053	502

* 314 swabs and 40 serum samples were collected from hunter-harvested WTD.

**Table 2:** SARS-CoV-2 whole-genome sequences from White-tailed deer.

Animal ID	Specimen	RT-qPCR Cq Value	Accession Number	Clade	Pango Lineage	Spike Mutations	Coverage
ZKOT6992	Oropharyngeal swab	25.2	EPI_ISL_19818736	24C	KP.3.2.8	S12F, A27S, L212I, F456L, Q493E	98%
OJLN7737	Oropharyngeal swab	15.0	EPI_ISL_19818737	24C	KP.3.2.8	S12F, A27S, L212I, F456L, Q493E	99.8%

**Table 3:** Positive samples for confirmatory SARS-CoV-2 spike ELISA.

Animal ID	Common Name	Scientific Name	Collection Date	City	VNT Titers	
					SARS-CoV-2 Omicron	SARS-CoV-2 -WA1/2020
24-1872	Eastern cottontail	*Sylvilagus floridanus*	19 June 2024	Sutton, MA	1:32	Not tested
24-0940	Eastern cottontail	*Sylvilagus floridanus*	16 May 2024	Auburn, MA	<1:16	<1:16
24-1313	Eastern cottontail	*Sylvilagus floridanus*	31 May 2024	Wrentham, MA	<1:16	<1:16
24-4074	Eastern cottontail	*Sylvilagus floridanus*	26 October 2024	Milford, MA	<1:16	<1:16
24-3041	Eastern coyote	*Canis latrans*	8 August 2024	Lexington, MA	<1:16	<1:16
24-3142	Eastern coyote	*Canis latrans*	13 August 2024	Framingham, MA	<1:16	<1:16
23-3009	Raccoon	*Procyon lotor*	28 August 2023	West Warren, MA	<1:16	<1:16
23-0738	Raccoon	*Procyon lotor*	8 May 2023	Westford, MA	<1:16	<1:16
24-4157	Raccoon	*Procyon lotor*	13 January 2024	Charlton, MA	<1:16	<1:16

## Data Availability

The data that support the findings of this study are available as Supplemental Material.

## Supplementary Materials

The following supporting information can be downloaded at: https://www.mdpi.com/article/10.3390/v18080832/s1. Table S1: List of free-ranging wildlife specimens collected between 2022 and 2025 in New England and Virginia and tested for SARS-CoV-2 RNA and antibodies.
